# Remarkable metabolic reorganization and altered metabolic requirements in frog metamorphic climax

**DOI:** 10.1186/s12983-020-00378-6

**Published:** 2020-10-08

**Authors:** Wei Zhu, Liming Chang, Tian Zhao, Bin Wang, Jianping Jiang

**Affiliations:** 1 CAS Key Laboratory of Mountain Ecological Restoration and Bioresource Utilization & Ecological Restoration Biodiversity Conservation Key Laboratory of Sichuan Province, Chengdu Institute of Biology, No.9, Section4, South Renmin Road, Chengdu, 610041 Sichuan China; 2 University of Chinese Academy of Sciences, Beijing, 100049 China

**Keywords:** Amphibian, Metabolic reorganization, Metabolic switch, Metamorphosis

## Abstract

**Background:**

Metamorphic climax is the crucial stage of amphibian metamorphosis responsible for the morphological and functional changes necessary for transition to a terrestrial habitat. This developmental period is sensitive to environmental changes and pollution. Understanding its metabolic basis and requirements is significant for ecological and toxicological research. *Rana omeimontis* tadpoles are a useful model for investigating this stage as their liver is involved in both metabolic regulation and fat storage.

**Results:**

We used a combined approach of transcriptomics and metabolomics to study the metabolic reorganization during natural and T3-driven metamorphic climax in the liver and tail of *Rana omeimontis* tadpoles. The metabolic flux from the apoptotic tail replaced hepatic fat storage as metabolic fuel, resulting in increased hepatic amino acid and fat levels. In the liver, amino acid catabolism (transamination and urea cycle) was upregulated along with energy metabolism (TCA cycle and oxidative phosphorylation), while the carbohydrate and lipid catabolism (glycolysis, pentose phosphate pathway (PPP), and β-oxidation) decreased. The hepatic glycogen phosphorylation and gluconeogenesis were upregulated, and the carbohydrate flux was used for synthesis of glycan units (e.g., UDP-glucuronate). In the tail, glycolysis, β-oxidation, and transamination were all downregulated, accompanied by synchronous downregulation of energy production and consumption. Glycogenolysis was maintained in the tail, and the carbohydrate flux likely flowed into both PPP and the synthesis of glycan units (e.g., UDP-glucuronate and UDP-glucosamine). Fatty acid elongation and desaturation, as well as the synthesis of bioactive lipid (e.g., prostaglandins) were encouraged in the tail during metamorphic climax. Protein synthesis was downregulated in both the liver and tail. The significance of these metabolic adjustments and their potential regulation mechanism are discussed.

**Conclusion:**

The energic strategy and anabolic requirements during metamorphic climax were revealed at the molecular level. Amino acid made an increased contribution to energy metabolism during metamorphic climax. Carbohydrate anabolism was essential for the body construction of the froglets. The tail was critical in anabolism including synthesizing bioactive metabolites. These findings increase our understanding of amphibian metamorphosis and provide background information for ecological, evolutionary, conservation, and developmental studies of amphibians.

## Introduction

Metamorphosis of amphibians often marks transition from a larval aquatic environment to a juvenile terrestrial environment [1]. This process is regulated by the interaction between thyroid hormones (THs: T3, the active TH form; T4, low-activity TH precursor) and its receptors [2–4]. In the morphological changes of tadpoles, metamorphosis is classified into pre-metamorphosis (stages 25–30; with no or rudimentary limb buds), pro-metamorphosis (stages 31–41; without external forelimbs), and metamorphic climax (stages 42–45; resorption of the tail) [1, 5]. Metamorphic climax is initiated by a peak of plasma T3 concentration in tadpoles [2, 3]. It involves dramatic changes in morphology and physiology, including remodeling of tadpole organs (e.g., the oral and gastrointestinal tract) into their adult form, resorption of tadpole-specific structures (e.g., the gill and tail), and development of adult-specific tissues such as limbs [6, 7]. It is a model system for studying the molecular network underlying the T3-mediated apoptosis, cellular reprogramming, and organogenesis in vertebrates [8–11]. Metamorphic climax is also a focus in ecology and toxicology studies of amphibians because it is a critical stage determining individual survival and population dynamics [12, 13].

Metabolism is at the end stage of cellular regulation cascades in response to endocrine signals and environmental factors [14]. The pivotal role of T3 in systematic metabolic regulation suggests comprehensive metabolic adjustments during the onset of metamorphic climax [15]. However, the metabolic requirements and adjustments potentially supporting the proceeding of morphological and functional transformation of metamorphic tadpoles are unclear [16, 17]. Energy metabolism is the basis of the many cellular processes. Adequate nutrient storage is essential for non-feeding metamorphic tadpoles to fuel their morphological remodeling and basic metabolism [7, 18–20]. Fat is the major fuel used during starvation in pro-metamorphic tadpoles [21, 22]. In tadpoles of some species (*Rana omeimontis* and *Xenopus laevis*), fat storage is also consumed during metamorphic climax [23], and its abundance provides a body condition signal to regulate the onset of metamorphic climax [21, 24]. The tail of tadpoles is another energy storage organ specific to metamorphic climax [25]. The tail and fat-storage organs (fat body and liver) may be complementary in supporting energy production and consumed sequentially during metamorphic climax [21, 22], but the coordination of metabolic fluxes from these storage organs is unclear.

The metabolic requirements of metamorphic tadpoles involve more than energy production. Organogenesis and organ remodeling rely on substantial biosynthesis [26]. The manner by which tadpoles coordinate the requirements of energy production and anabolism with limited nutrient storage is unknown, but may help further the understanding of the mechanisms determining froglet body size. Individual development and organogenesis appear to be coupled with a switch of metabolic substrates and reorganization of the metabolic network [27–30]. All three major types of metabolic substrates (carbohydrates, lipids, and amino acids) can be used as fuel for energy production, but their roles in metabolite interconversion and biosynthesis appear to be different [31]. This implies metabolic reorganization during the onset of metamorphic climax. The encouraged metabolic pathways may be critical for the metamorphic climax process. It is also important to determine if metabolites help to regulate metabolic coordination or the metamorphic processes.

**Fig. 1:**
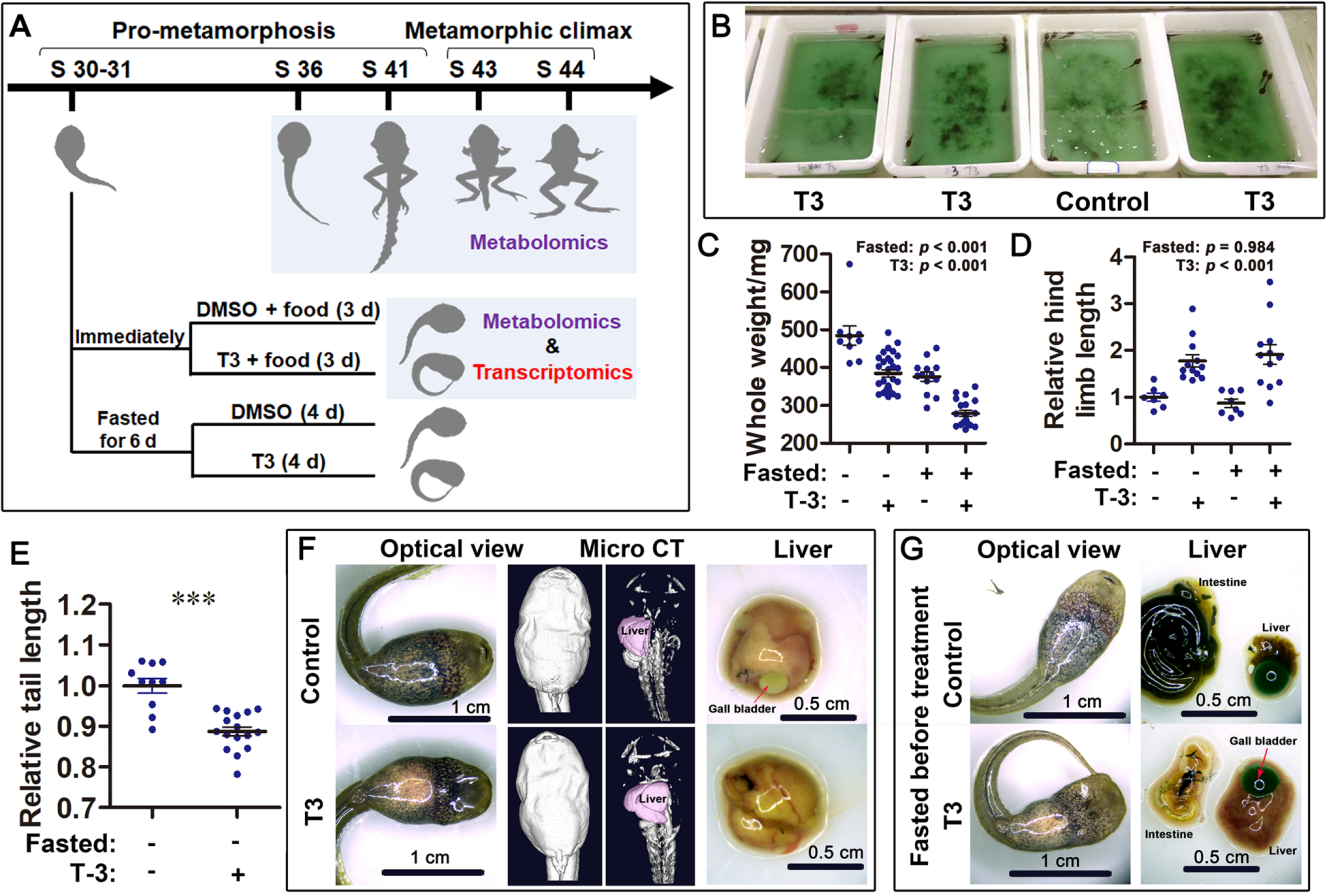
Experimental design and T3-driven metamorphic climax. **a** Experimental design. **b**–**g** T3-induced morphological and physiological changes in pro-metamorphic *R. omeimontis* tadpoles (stages 30–31). T3-treated tadpoles had reduced food intake (**b**), reduced body weight (**c**), accelerated development of hind limbs (**d**), shortened tail (**e**), broadened oral disk width (**f**–**g**), and reduced mobilization of hepatic resources (**f**–**g**; reflected by the liver size and morphology). Food intake was reflected by the residual content of spirulina powder in the water; the higher content of the spirulina powder, the darker the green color of the water. *p* < 0.001

To study the metabolic requirements of metamorphic climax, it is useful to examine the systematic adjustments of metabolic fluxes within and between major metabolic organs. The liver plays a central role in regulating metabolism. It coordinates the metabolite fluxes from different energy storage organs (fat body and the tail). The metabolic adjustments in the liver and energy storage organs, as well as the metabolic interactions between organs, likely illustrate the dynamic changes in metabolism during metamorphic climax. *Rana omeimontis* tadpoles lack fat-accumulating fat bodies across their larval stages, and the liver serves as their primary fat storage organ [21]. This characteristic simplifies analyses on the metabolic flexes across organs. In addition, the metabolic pattern during the fasting period has been studied in *R. omeimontis* tadpoles [21]. This information highlights the metabolic adjustments specific to metamorphosis. In this study, we reconstruct and compare the metabolic networks in the tail and the liver between pro-metamorphic and metamorphic (natural or T3-driven) *R. omeimontis* tadpoles. We used a combination of comparative transcriptomics and metabolomics (Fig. 1a).

## Materials and methods

### Animal culture

Ten clutches of *R. omeimontis* were collected in October at the Anzihe Natural Reserve (103.459885° E, 30.744614° N, 701 m) in Sichuan Province, China. The laboratory conditions for egg hatch and tadpole culture followed the methods described by Zhu et al. [21]. After hatching, tadpoles from the same clutch were divided into several populations with population size ranging from 400 to 1000 individuals. These populations were fed with ground spirulina powder (China National Salt Industry Corporation, Tianjin, China) daily following the protocol of Zhu et al. [21]. In control experiments, all the tadpole groups were provided with the same amount of food. The spirulina powder gives the water a green color, and water with a darker green color indicates more residual food. This character facilitated the comparison of food intake between groups.

### Experimental design

To study the metabolic adjustments associated with metamorphic climax, we designed two experimental systems (Fig. 1a). First, *R. omeimontis* tadpoles were collected at their pro-metamorphic stages (Gosner stages 36 and 41) and metamorphic stages (stages 43 and 44), respectively [5]. Their liver (stages 36, 41, 43, and 44) and tail (stages 36, 41 and 43) were sampled for metabolic profiling, and comparative analyses across stages were conducted to reveal the metabolic change specific to the onset of metamorphic climax. Second, *R. omeimontis* tadpoles were collected at stages 30–31 and treated with exogenous dimethylsulfoxide (DMSO) or T3 to obtain a pro-metamorphic group and a metamorphic group, respectively [32]. Tadpoles at stages 30–31 were randomly divided into two groups and treated with DMSO (control) or 10 nM T3 in plastic containers (20 × 15 × 8 cm, with 600 mL water), until typical climax metamorphic traits (e.g., unbalanced swimming, tetanic and shortened tail, tetanic hind limbs, and broadened oral disk) were observed in the T3-treated group. Three days of treatment was required, and spirulina powder was provided constantly during treatment (Fig. 1a). A series of behavioral, morphological, and physiological indexes were measured to assess the availability of T3 in imitating metamorphic climax. Then, the two groups were compared for their metabolome and transcriptome in the liver and tail. We found that the effect of T3 on mobilization of hepatic fat may be disturbed by the different feeding activity between T3 and DMSO treatment groups, as well as by their level of hepatic fat. Thus, another two groups of stage 30–31 tadpoles were starved for 6 days before treatment to reduce the fat content in their liver. In this test, 4 days of treatment was required to observe obvious metamorphic traits in T3 group, because the presence of food may have promoted consumption of T3. In this study, the term “metamorphic tadpole” may either refer to the tadpoles at their natural metamorphic climax or T3-treated pro-metamorphic tadpoles (T3-driven metamorphic climax).

### Micro-computed tomography

A micro-computed tomography (Micro-CT) scan was used to examine the morphological variation of the liver during metamorphic climax. After anesthetization by MS-222, tadpoles were fixed in 4% paraformaldehyde for more than 24 h and stained in I_2_ & KI water solutions (respectively, 1% & 2%, w/v) for 12 h [21]. A Micro-CT scan was conducted on a Quantum GX Micro CT (PerkinElmer, Waltham, MA, USA) with the following parameters: scanning current, 70 eV; 10 μM; field-of-view: 36 × 36 mm for acquisition, 25 × 25 mm for reconstruction; scan duration, 15 min.

### Histological section

Histological sections were made to study the morphological changes of hepatocytes and their fat content. Liver samples were collected and fixed in 4% paraformaldehyde. Tissue slices were prepared using the method of Wang et al. [33]. Hematoxylin and eosin (H&E) staining and Oil Red O (ORO) staining were conducted to show general histological characteristics and neutral lipid content, respectively.

### Metabolomic analysis

Comparative metabolomics of the tail and liver was conducted between T3 and DMSO-treated tadpoles, as well as between natural metamorphosing tadpoles at different stages. After grinding in liquid nitrogen, every 100 mg liver or tail (*n* = 6 for each natural developmental stage; 7 and 10 for control and T3 treatment groups, respectively) powder was transferred into 1.5 mL Eppendorf tubes with 1 mL methanol: acetonitrile: water = 2: 2: 1 (v/v), and the metabolites were extracted following the methods described by Zhu et al. [21]. Extracted supernatants were analyzed by LC (1290 Infinity LC, Agilent, Santa Clara, CA, USA) coupled with quadrupole time-of-flight mass spectrometry (Triple TOF 5600+, AB SCIEX). The chromatographic parameters and programs, as well as the mass spectrum parameters, followed the methods described by Zhu et al. [21].

Metabolite data were processed using XCMS software (http://metlin.scripps.edu/download/) and Microsoft Excel (Microsoft, Redmond, WA, USA). Metabolites were identified by a combination of molecular weight comparison (molecular ion peak) and MS/MS spectrum comparison to a standard library. The relative abundances/concentrations of metabolites are presented as the ion intensities of their molecular ion peaks (Additional file 1).

### Transcriptomic analysis

Comparative transcriptomics of the tail and liver was conducted in T3 and DMSO-treated tadpoles. Total RNA of each liver or tail sample (*n* = 3 for each stage or treatment group) was extracted using a TRIzol kit (Invitrogen, Carlsbad, CA, USA), following manufacturer instructions. After RNA quantification, quality assessment and purification, cDNA libraries were built following the methods described by Zhu et al. [34]. After cluster generation, the libraries were sequenced on an Illumina HiSeq 2500 platform by Annoroad (Beijing, China), and paired-end reads were generated. The clean reads were obtained from raw reads by removing the adapter reads, as well as poly-N and low-quality reads. All clean reads were assembled de novo using Trinity as the reference transcriptome. The resulting unigenes were annotated by querying against NR database with an E-value threshold of 1.0e^− 5^. Then, the FPKM values of each unigene in samples were calculated by Bowtie and RSEM (see transcriptome processing results in Additional file 2: Figure S1). Sequencing data from this study was submitted to the NCBI Gene Expression Omnibus (GEO; http://www.ncbi.nlm.nih.gov/geo/) under accession number GSE147618.

### Statistical analyses

Statistical analyses were done using IBM SPSS v21.0 (SPSS Inc., Chicago, IL, USA). The effects of T3 treatment on morphological traits were analyzed using independent sample *T* tests (relative tail length) or a mixed model ANOVA (body weight and relative hind limb length). Variations in metabolite and gene expression levels between the groups were evaluated by independent sample t tests or one-way ANOVA and Student–Newman–Keuls post hoc tests. Principal component analysis (PCA) of metabolomes was conducted using Simca-P + 11 (Umetrics AB, Umea, Sweden), with the scaling-type parameter set as ‘Par’. Graphs were created using GraphPad Prism 5 or ggplot2, an R package [35].

## Results

T3 treatment reduced the food intake of *Rana omeimontis* tadpoles (Fig. 1b). After 3–4 d of treatment, these tadpoles had decreased weight (Fig. 1c), accelerated hind limb development and tail absorption (Fig. 1d–e), and broadened oral disk width (Fig. 1f). In contrast to the increased consumption of hepatic resource in starved pro-metamorphic tadpoles [21], T3-treated tadpoles had a liver size similar to the control group despite their reduced food intake (Fig. 1f and Additional file 2: Figure S2). When food was not provided during treatment, T3-treated tadpoles had larger livers than the control group (Fig. 1f and Additional file 2: Figure S2).

### Dramatic metabolic reorganization during onset of metamorphic climax

**Fig. 2:**
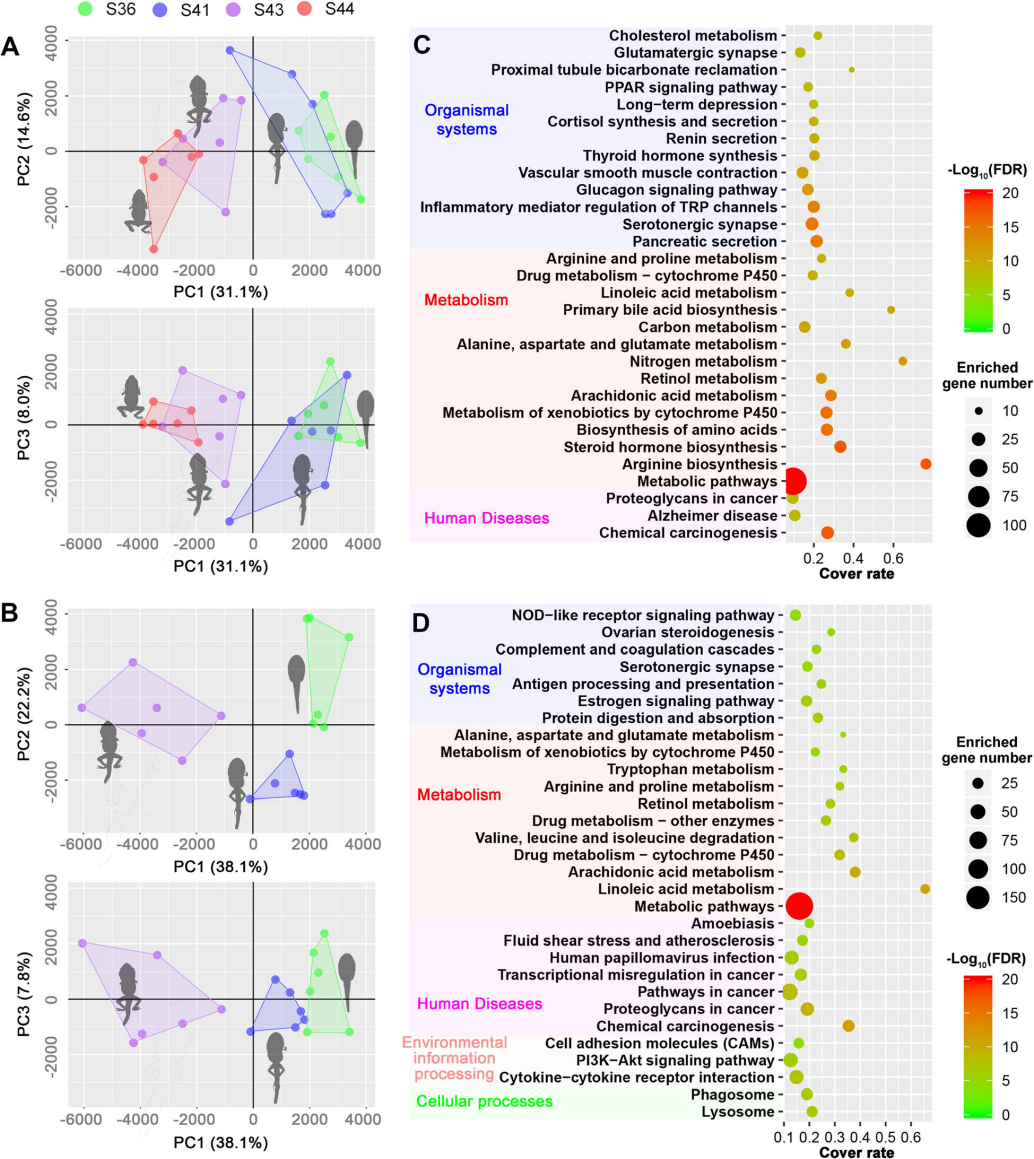
Dramatic metabolic reorganization during metamorphic climax. **a** and **b** Scatter plots of PCAs based on liver (**a**) and tail (**b**) metabolomes of tadpoles at different Gosner stages (*n* = 6 for each organ at each stage). **c** and **d** Top 30 significantly enriched KEGG pathway based on liver (**c**) and tail (**d**) DEGs between T3-treated and control tadpoles. The pathway categories were adapted from the KEGG pathway database. The cover rate is the ratio between number of genes enriched in a pathway and the total number of genes in this pathway

Metamorphosis from pro-metamorphic to metamorphic stages was associated with dramatic metabolic adjustments. The variation of liver and tail metabolomes divided tadpoles into pro-metamorphic (stages 36 and 41) and metamorphic groups (stages 43 and 44) along the first primary component (PC1, accounting for 31.1% of the total variance) of PCA (Fig. 2a–b and Additional file 2: Figure S3; see detailed metabolomic data in Tables S1, S2, S3, S4). At transcriptional level, KEGG enrichment analyses were conducted for DEGs between T3-treated and control groups (Additional file 2: Figure S1). Metabolic pathways accounted for the largest proportion of the top 30 significantly enriched items (14/30 for the liver and 11/30 for the tail; Fig. 2c–d; Table S5–S6).

### Lipid metabolism in the liver during metamorphic climax

**Fig. 3:**
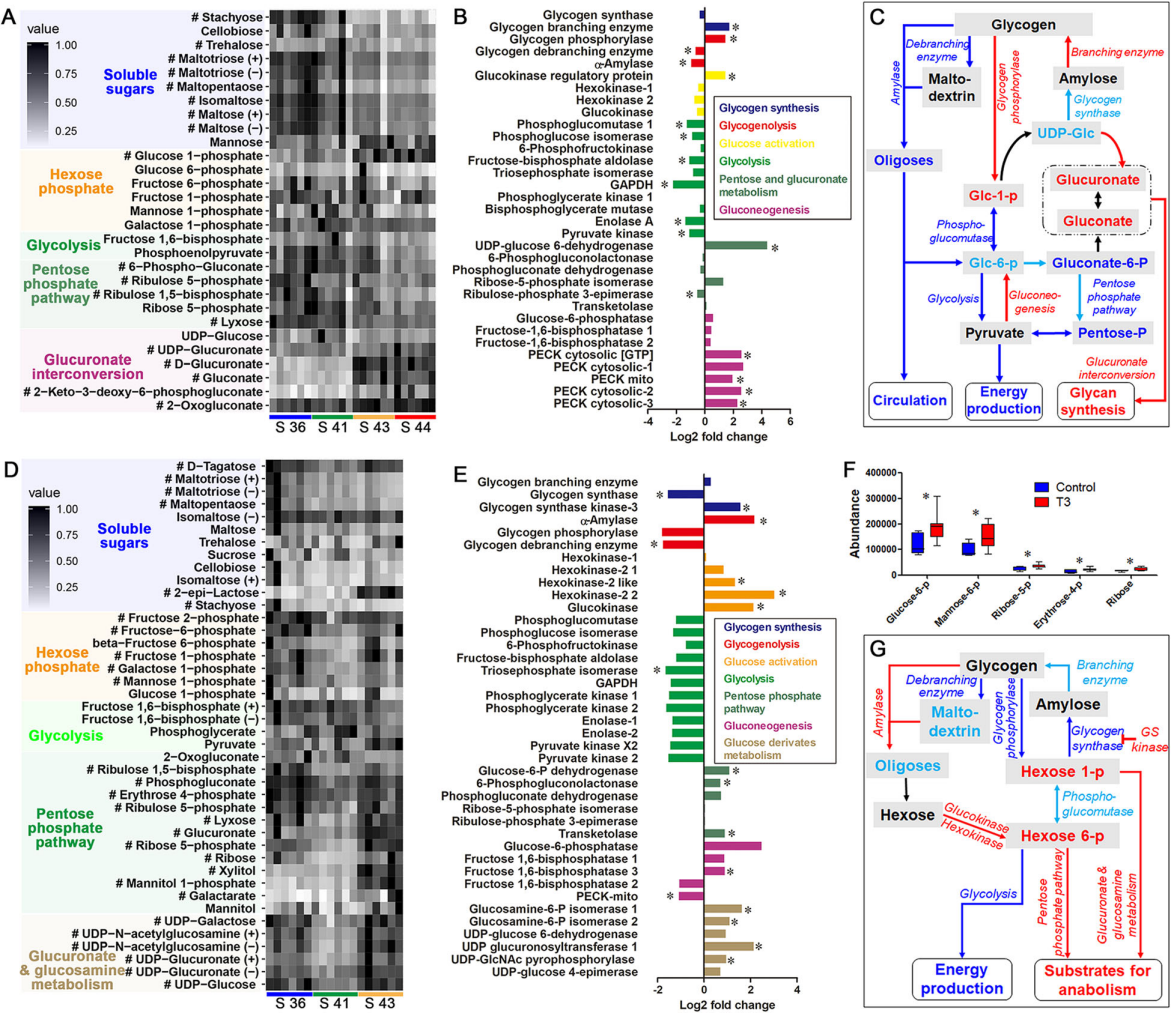
Reorganization of lipid metabolism in the liver during metamorphic climax. **a**–**b** Free fatty acids (FAAs) and acylcarnitines varied (*p* < 0.05, one-way ANOVA) during natural metamorphosis. Different letters denote significant differences between groups (*p* < 0.05), as shown by the Student–Newman–Keuls post hoc test after one-way ANOVA. **c** FFAs and acylcarnitines differed in content between control and T3-treated groups. Each box represents a mean ± SE; *, *p* < 0.05. **d** Transcriptional changes of genes involved in lipid metabolism in the liver after T3-treatment; a positive log-transformed fold change value means upregulation in T3-treated group, and vice versa; *, *p* < 0.05. **e** Histological sections of the liver. Triacylglycerol (TAG) is the major form of hepatic fat storage in the liver and accounts for the red color in Oil Red O (ORO) staining. **f** Network presenting the adjustments on lipid metabolism in the liver. Metabolic fluxes are presented as arrows between items. Items and arrows with blue, red, cyan, and black colors indicate downregulated/decreased, upregulated/increased, unchanged, and undetected, respectively; and similarly hereinafter

With the progression of metamorphosis from stage 36 to stage 44, four free fatty acids (FFAs; Δ18:1, Δ18:3, Δ16:0, and Δ16:1) and two aryl-carnitines (Δ18:0-carnitine and Δ10:0-carnitine; active form of FFAs) decreased and increased in content, respectively (*p* < 0.05, one-way ANOVA; Fig. 3a–b). Levels of other FFAs and aryl-carnitines were unchanged. Three FFAs (Δ18:1, Δ18:2, and Δ16:0) and two acyl-carnitines (Δ16:0-carnitine and Δ18:0-carnitine) decreased and increased in content, respectively, in T3-treated groups (Fig. 3c). Other FFAs or acyl-carnitines were unaffected. At the transcriptional level, T3-treated tadpoles showed upregulated lipogenesis (diacylglycerol/DAG O-acyltransferases), but downregulated lipolysis (hepatic triacylglycerol/TAG lipase and DAG lipase), fatty acid transport (fatty acid binding protein and long-chain fatty acid transport protein), FFA β-oxidation (acyl-CoA dehydrogenase and trifunctional protein), and other types of FFA oxidations such as fatty aldehyde dehydrogenase and fatty acid 2-hydroxylase (Fig. 3d). Correspondingly, cholesterol synthesis, the downstream pathway of FFA oxidation, was also downregulated at the transcriptional level (Fig. 3d). Bile acid and steroid hormone metabolism, the catabolic routes of cholesterol, were also downregulated (Additional file 2: Figure S4). Histological sections indicated that T3-treated tadpoles contained more hepatic fat (larger vacuoles in H&E staining and larger red area in red oil staining) than the control group (Fig. 3e). Taken together, these results suggest that hepatic fat consumption was reduced after the onset of metamorphic climax, and the fatty acid flux in the liver was encouraged to flow into TAG synthesis rather than degradation and sterol synthesis (Fig. 3f). The liver of metamorphic tadpoles showed decreased expression of peroxisome proliferators-activated receptor alpha (PPARα) (Additional file 2: Figure S5).

### Lipid metabolism in the tail during metamorphic climax

**Fig. 4:**
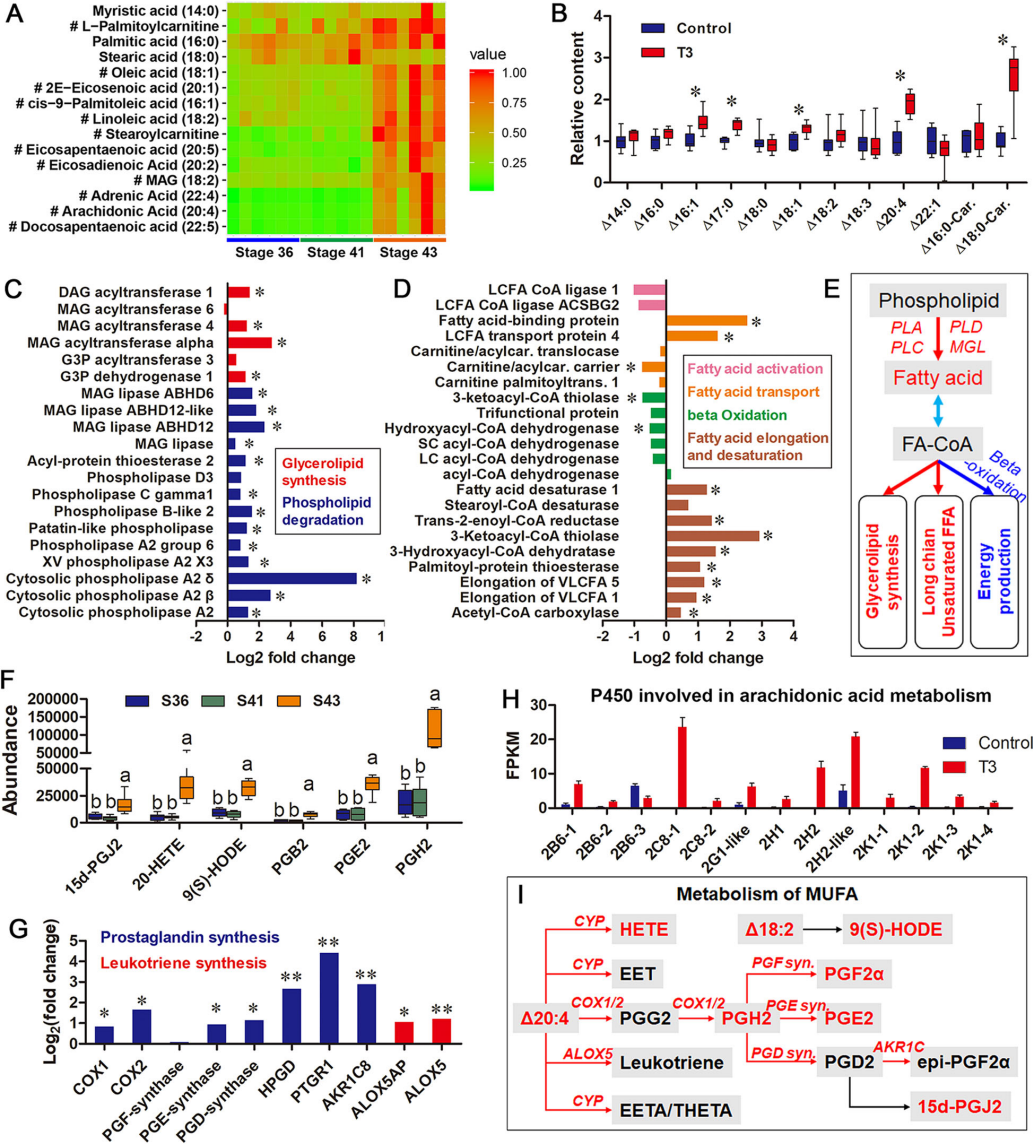
Reorganization of lipid metabolism in the tail during metamorphic climax. **a** Heatmap showing the variation of fatty acids during natural metamorphosis; #, *p* < 0.05 (one-way ANOVA). **b** FFAs and acylcarnitines differed in content between control and T3-treated groups; *, *p* < 0.05. **c**–**d** Transcriptional changes of genes involved in lipid metabolism after T3-treatment; *, *p* < 0.05. **e** Network denotes adjustments on lipid metabolism in the tail. **f** FFA derivatives/bioactive lipids varied in content (*p* < 0.05, one-way ANOVA) during natural metamorphosis (PG, prostaglandin; HETE, hydroperoxyeicosatetraenoic acid; HODE, hydroxyoctadecadienoic acid). Different letters denote significant differences between groups (*p* < 0.05), as determined by the Student–Newman–Keuls post hoc test after one-way ANOVA. **g** Transcriptional changes of critical enzymes involved in prostaglandin and leukotrienes biosynthesis. *, *p* < 0.05; **, *p* < 0.01. (H) Differently expressed cytochrome P450 genes involved in arachidonic acid metabolism (*p* < 0.05). (I) Network denotes the upregulated synthesis of FFA derivatives

Metamorphosis from pro-metamorphic stages (stages 36 and 41) to metamorphic climax (stage 43) was accompanied by a dramatic increase of most unsaturated FFAs, acyl-carnitines, and MAG (Fig. 4a). T3 treatment partly reproduced these metabolic changes in pro-metamorphic tadpoles by inducing the levels of Δ16:1, Δ17:0, Δ18:1, Δ20:4, and Δ18:0-carnitine (Fig. 4b). At the transcriptional level, T3-treated tadpoles showed upregulated glycerolipid synthesis (DAG/MAG O-acyltransferase), phospholipid degradation (phospholipases), and fatty acid elongation and desaturation (fatty acid desaturases and elongation of long chain fatty acid proteins), but downregulated FFA β-oxidation (2-ketoacyl-CoA dehydrogenase and hydroxyacyl-CoA dehydrogenase) (Fig. 4c–d). These results suggest an accelerated degradation of phospholipids during metamorphic climax. The resulting FFAs flux was mainly diverted to synthesis of glycerolipid and long-chain unsaturated fatty acid, rather than to further catabolism (Fig. 4e). T3-treated tadpoles had increased transcription of PPARα, PPARβ, and PPARγ in their tail (Additional file 2: Figure S5). Their tail also showed decreased transcription of adiponectin, a secretory metabolic regulator, while the transcription of adiponectin receptors was upregulated (Additional file 2: Figure S5).

The tails of metamorphic tadpoles showed accumulation of prostaglandins (PGs) and hydroperoxyeicosatetraenoic acid (HETE) (Fig. 4f) that are derivatives of unsaturated FFAs. T3 treatment upregulated arachidonic acid metabolism (PG synthases and cytochrome P450) (Fig. 4g–i and Additional file 2: Figure S6), which was responsible for synthesizing these derivatives. These results suggest increased synthesis of functional FFA derivatives in the tail during metamorphic climax.

### Carbohydrate metabolism in the liver during metamorphic climax

**Fig. 5:**
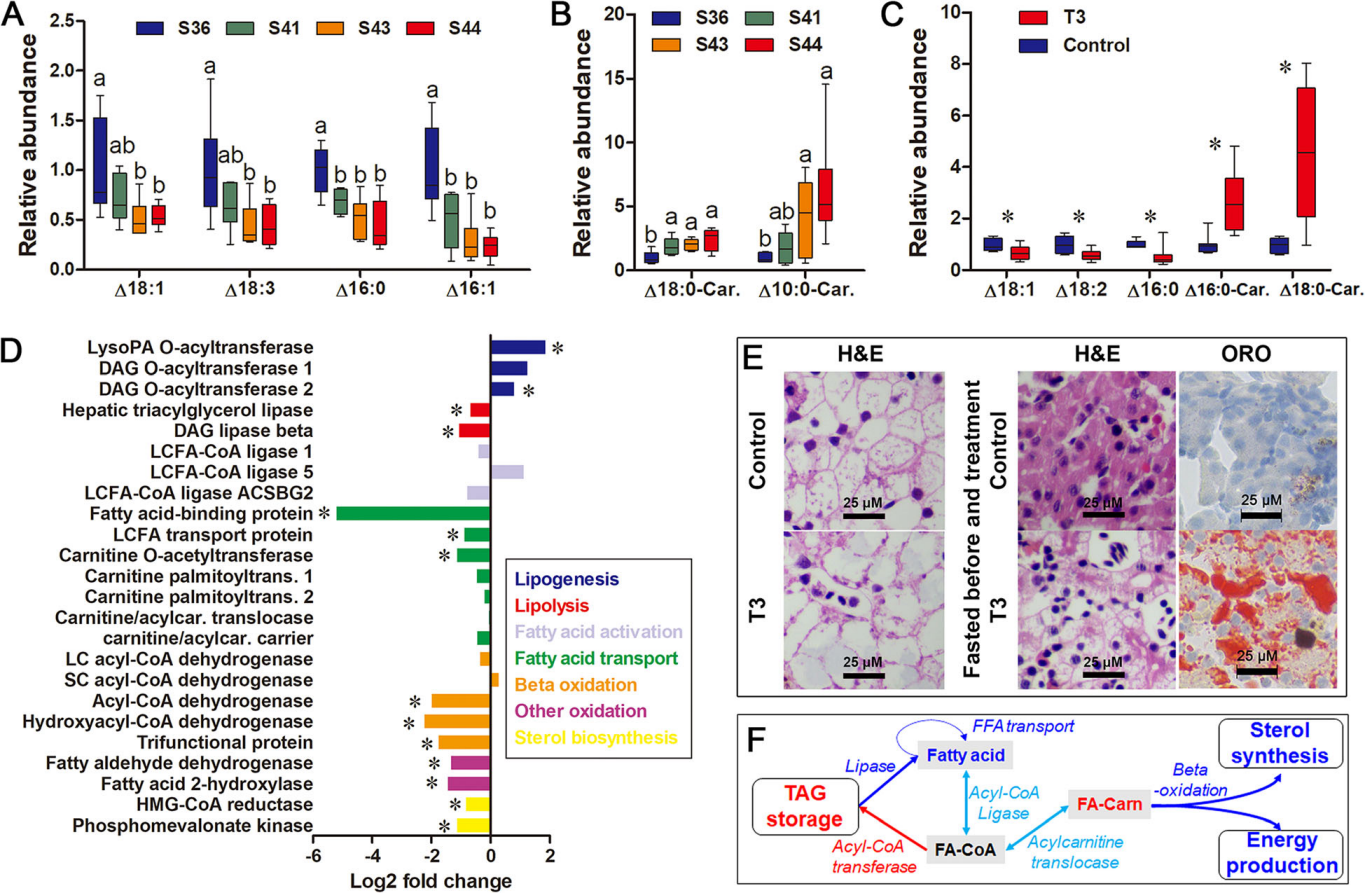
Reorganization of carbohydrate metabolism during metamorphic climax. **a**–**c** Carbohydrate metabolism in the liver. **a** Heatmap showing the variation of metabolites of glycol-metabolism when metamorphosis proceeds from stage 36 to stage 44, #, *p* < 0.05 (one-way ANOVA). **b** Transcriptional changes of genes involved in carbohydrate metabolism in the liver after T3-treatment; a positive log-transformed fold change value means upregulation in T3-treated group, and vice versa; *, *p* < 0.05. **c** Network denotes adjustments on lipid metabolism in the liver. (D–G) Carbohydrate metabolism in the tail. **d** Heatmap showing the variation of metabolites of glycol-metabolism when metamorphosis proceeds from stage 36 to stage 43, #, *p* < 0.05 (one-way ANOVA). **e** Transcriptional changes of genes involved in carbohydrate metabolism in the tail after T3-treatment; a positive log-transformed fold change value means upregulation in T3-treated group, and vice versa; *, *p* < 0.05. **f** Variation of metabolites in the pentose phosphate pathway (PPP) after T3-treatment. Each box represents the mean ± SE; *, *p* < 0.05. **g** Network denotes adjustments on lipid metabolism in the tail

Metamorphic tadpoles (stages 43 and 44) had decreased levels of hepatic disaccharides and trisaccharides (the major forms of soluble sugar in the liver of *R. omeimontis* tadpoles such as maltose and maltotriose) (Fig. 5a). Consistent with that, T3-treated tadpoles had downregulated transcription of glycogen debranching enzyme and α-amylase in their liver (Fig. 5b). This suggests reduced mobilization of glycogen through hydrolyzation. Metamorphic tadpoles showed increased transcription of glycogen phosphorylase and an increased level of glucose 1-phosphate (Fig. 5a–b), suggesting that glycogen mobilization was maintained through the phosphorylation route during metamorphic climax. Phosphoglucomutase was the critical enzyme diverting glucose 1-phosphate to glycolysis and PPP by converting it to glucose 6-phosphate. In the liver of metamorphic tadpoles, its downregulation and decreased transcription of enzymes involved in glycolytic enzymes (phosphoglucose isomerase and fructose-biphosphate aldolase) suggested reduced metabolic fluxes throughout glycolysis (Fig. 5b–c), even though the level of glycolytic intermediates (hexose 6-phosphates and fructose 1,6-biphosphate) was maintained (Fig. 5a). This was consistent with the increased transcription of phosphoenolpyruvate carboxykinases (PECKs) (Fig. 5b), the critical enzymes of gluconeogenesis. Similarly, metamorphic tadpoles had decreased levels of PPP intermediates (gluconate 6-phosphate and ribulose 5-phosphate) and downregulated transcription of ribulose-phosphate 3-epimerase (Fig. 5b–c), suggesting reduced metabolic flux throughout PPP during metamorphic climax. UDP-glucose 6-dehydrogenase is responsible for converting glucose 1-phosphate to UDP-glucuronate. Its upregulated transcription and increased levels of UDP-glucuronate and related metabolites in metamorphic tadpoles suggests that glucuronate interconversion was enhanced and likely responsible for the increased metabolic flux from glycogen to glucose 1-phosphate (Fig. 5a–c).

### Carbohydrate metabolism in the tail during metamorphic climax

Because the two pro-metamorphic stages (stages 36 and 41) differed in their tail profiles of glycolytic metabolites (Fig. 5d), our analyses focused on the differences between stage 41 and 43 to highlight the metabolic changes associated with the onset of metamorphic climax. The levels of disaccharides and trisaccharides (maltotriose and maltopentaose) in their tail was maintained when metamorphosis proceeded from stage 41 to 43 (Fig. 5d). Although the transcription of glycogen debranching enzyme was downregulated in metamorphic tadpoles, their transcription of α-amylase was upregulated (Fig. 5e). These results suggest that glycogen mobilization in the form of disaccharides and trisaccharides was maintained. The decreased transcription of glycogen phosphorylase suggests reduced mobilization of glycogen through phosphorylation (Fig. 5e). The increased transcription of glycogen synthase and decreased glycogen synthase kinase suggests that glycogen synthesis was suppressed during metamorphic climax (Fig. 5e). Metamorphic tadpoles (stage 43) maintained higher levels of hexose phosphates (fructose 1-phosphate and fructose 6-phosphate) than pro-metamorphic tadpoles (stage 41) (Fig. 5d). This was consistent with the increased transcription of hexokinases and glucokinase in T3-treated tadpoles (Fig. 5e), suggesting increased metabolic flux from soluble sugar (glycogenolysis and tissue apoptosis) to hexose phosphates. This carbohydrate flux was not likely diverted into glycolysis, as the transcription of glycolytic enzymes (triosephosphate isomerase) was downregulated by T3 treatment (Fig. 5e). In contrast, PPP and glucuronate and glucosamine metabolism were likely encouraged during metamorphic climax, as metamorphic tadpoles maintained increased levels of intermediates in PPP and glucose derivates (glucuronate and glucosamine) (Fig. 5d and f), as well as increased transcription of related enzymes (Fig. 5e). These results suggest that the carbohydrate flux in the tail was preferentially allocated to metabolic shunts associated with biosynthesis, rather than energy production (Fig. 5g).

### Protein and amino acid metabolism during metamorphic climax

**Fig. 6:**
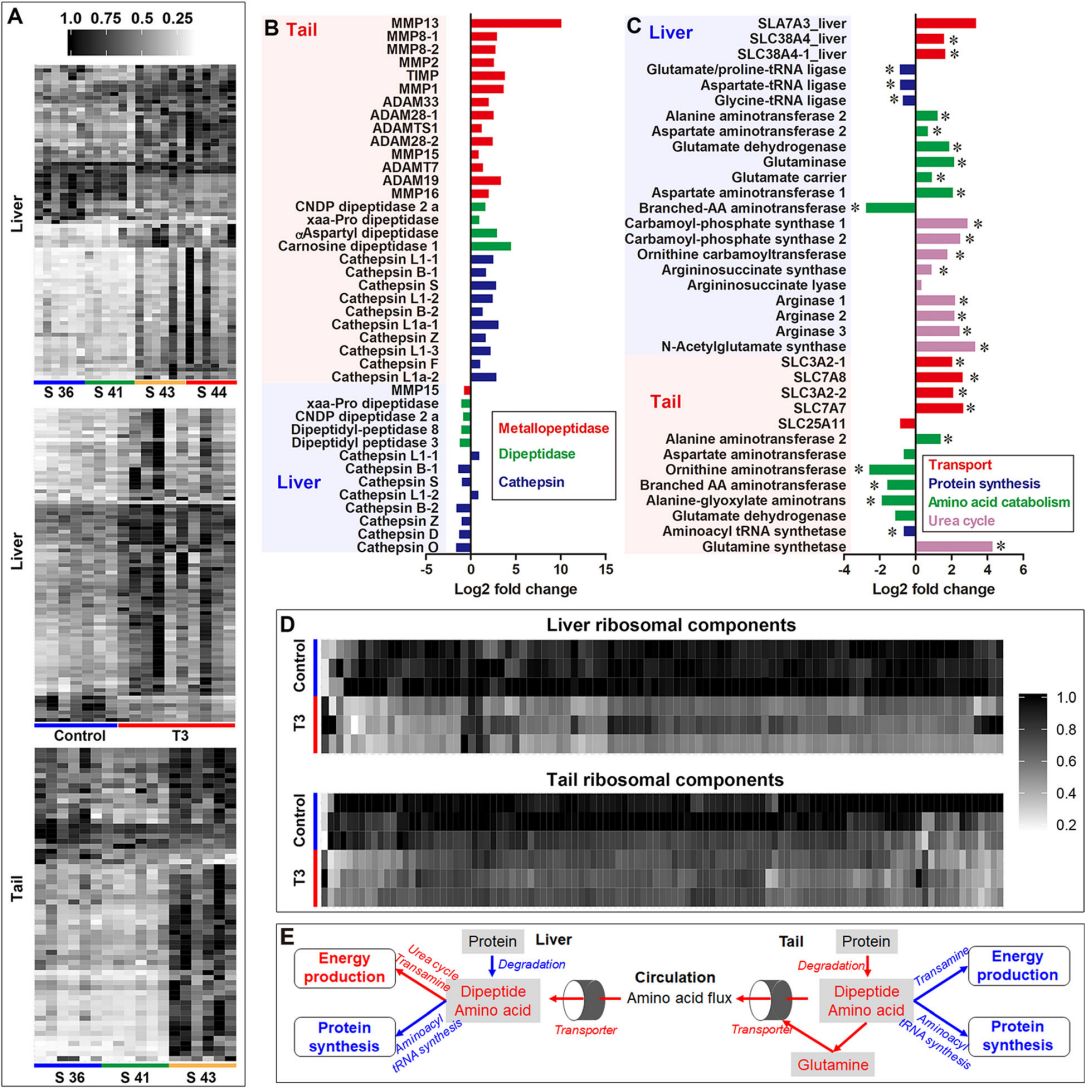
Amino acid metabolism during metamorphic climax. **a** Variation of amino acids and dipeptide during natural and T3-driven metamorphic climax. **b**–**c** Transcriptional changes of genes involved in amino acid metabolism in the liver after T3-treatment; a positive log-transformed fold change value means upregulation in the T3-treated group, and vice versa; *, *p* < 0.05. **d** Heatmap showing the variation of ribosomal components (FPKM > 50) during natural metamorphosis. **e** Network denotes adjustments on amino acid metabolism

Amino acids and dipeptides increased in the liver and tail after the onset of metamorphic climax (Fig. 6a). T3-treatment induced increased transcription of metallopeptidases, dipeptidases, and cathepsins in the tail, but not in the liver (Fig. 6b), while increased transcription of amino acid transporters was observed in both the liver and tail (Fig. 6c). These results suggest accelerated protein degradation in the tail and increased amino acid flux from the tail to liver during metamorphic climax.

In the liver, metamorphic tadpoles showed decreased transcription of aminoacyl-tRNA synthetases and ribosomal components but increased transcription of aminotransferases and enzymes of urea cycle (carbamoyl-phosphate synthase and argininosuccinate lyase) (Fig. 6c–d). This suggested that amino acid catabolism increased in the liver, rather than protein synthesis, during metamorphic climax (Fig. 6e).

In the tail, the transcription of aminoacyl-tRNA synthetase, ribosomal components, and most aminotransferases was downregulated (Fig. 6c–d), suggesting simultaneous suppression of amino acid catabolism and protein synthesis during metamorphic climax (Fig. 6e). T3 treatment increased the transcription of glutamine synthetase in the tail (Fig. 6c), and the levels of glutamine increased in both tail and liver of metamorphic tadpoles (Additional file 2: Figure S7). This suggested that tail ammonia was recycled in the form of glutamine during metamorphic climax (Fig. 6e).

### TCA cycle and oxidative phosphorylation during metamorphic climax

TCA cycle and oxidative phosphorylation are common downstream processes of lipid, carbohydrate, and amino acid catabolism. Metamorphic tadpoles showed an overall upregulated transcription of these two pathways in their liver, but downregulated transcription in their tail (Additional file 2: Figure S8 A–C). In the tail, Ca^2+^-ATPases and muscle creatine kinases, the primary ATP consumers in muscle, were also downregulated, while the transcription of uncoupling protein (UCP), which diverts proton gradient away from energy production, was upregulated (Additional file 2: Figure S8 D).

## Discussion

Our results show that the onset of metamorphic climax in *R. omeimontis* tadpoles was associated with dramatic metabolic changes in the liver and tail (Figure 2). Using naturally metamorphosing tadpoles (stages 30–31, 36, 41, 43, and 44) and T3-driven metamorphic tadpoles as model systems, we studied the metabolic adjustments systematically by reconstructing the primary metabolic pathways of carbohydrate, lipid, and amino acids based on comparative transcriptomics and metabolomics. The significance of these adjustments in sustaining the proceeding of metamorphic climax are discussed below.

### Mobilization of nutrient storage

During metamorphic climax, energy metabolism in the liver of *R. omeimontis* tadpoles was upregulated at the transcriptional level (Additional file 2: Figure S8 A). This could be due to the increased requirement of biosynthesis and metabolite interconversion during metamorphic climax [36]. Despite of the accelerated energy consumption, the liver mass and hepatic storages of metamorphic tadpoles were not decreased (Fig. 1f; Zhu et al. [21]). Even their feeding behavior had stopped during metamorphic climax (Fig. 1b). This suggests that extra-hepatic nutrients were mobilized, as fuel, to replace the hepatic storage. The tail is the largest organ undergoing apoptosis during metamorphic climax. It undergoes accelerated degradation of cellular components during metamorphic climax (Fig. 6b), with synchronously downregulated energy production and consumption (Additional file 2: Figure S8 B–D). These metabolic changes suggest that it transformed from a primary energy consuming organ to nutrient storage. Amino acids are likely the primary type of metabolic substrates flowing from the tail to the liver (Fig. 6e). The increased transcription of UCP in the tail (Additional file 2: Figure S8 D) suggests that this organ was not energy deficient during metamorphic climax [37], which might be important to nutrient recycling from the tail.

Although the tail could replace the fat depot as an energy resource after the onset of metamorphic climax, starvation might last longer than tail apoptosis due to physiological or environmental constraints [12]. Fat storage, in the fat body or liver, may assume the responsibility of nutrient supply once the tail has been resorbed into a stub [21, 22]. This means that both the tail and fat storage (fat body or liver) are necessary and mobilized sequentially to complete metamorphosis.

### Metabolic shift in energy metabolism

At the molecular level, the liver of metamorphic *R. omeimontis* tadpoles had upregulated amino acid catabolism (transamination and urea cycle) (Fig. 6e) while its FFA β-oxidation and glycolysis were downregulated (Figs. 3f and 5c). In combination with the increased energy metabolism in the liver (Additional file 2: Figure S8 A), these results suggest a metabolic shift from carbohydrate and lipid to amino acids in energy metabolism in the liver during metamorphic climax. This is different from the metabolic pattern in the starved pro-metamorphic tadpoles, who preferentially consumed hepatic lipid and glycogen [21]. Why were amino acids preferentially consumed by catabolism during metamorphic climax? First, the requirement of the three types of substrates in building froglets might differ. The upregulated gluconeogenesis in the liver (Fig. 5c) suggests that apoptotic tissue might provide insufficient carbohydrates to sustain related anabolism. This is reasonable, as carbohydrate is required in synthesizing nucleotide and glycan and for providing most NADPH [31]. In contrast, the increased glycerolipid synthesis (Fig. 3f) and ammonia deposition (Fig. 6c) in the liver suggested that amino acids and lipids recovered from tissue apoptosis likely exceeded the amount required for synthesizing froglet proteins and phospholipids. Second, organ remodeling and organogenesis require synthesis of large amounts of cellular components. In comparison to lipids, amino acids can provide more types of carbon skeletons through incomplete oxidation and this might satisfy the requirement of energy production and metabolite interconversion simultaneously. Third, although lipids and amino acids may be equivalent as energy fuel, amino acids or proteins are less convenient for bulk storage than lipids. As the non-feeding period may last longer than the duration of tissue apoptosis during metamorphic climax, tadpoles may be unable to store sufficient energy in amino acids for the late metamorphic climax. This means that these two nutrients are mobilized to sustain different metabolic requirement of metamorphosis. It is possible that the tail size and fat amount might be independent body condition signals for the onset of metamorphic climax. The former is more likely to be correlated with the froglet body size.

### Anabolic requirements during metamorphic climax

Shifting anabolic requirements are expected during the transition from fat-growing stages to metamorphic climax. Amphibian metamorphic climax is characterized by a shrinkage of the body size. This indicates that some constitutive cellular components could be recovered from apoptotic tissues, and not all of the anabolic processes are maintained. A typical example is cholesterol, a necessary cellular component limiting cell growth [38]. During metamorphic climax, cholesterol synthesis was downregulated in the liver (Fig. 3f). Its catabolism (hydroxylation and de hydroxylation) was also downregulated (Additional file 2: Figure S4). This suggests a reduced turnover rate of cholesterol in metamorphic tadpoles, which might be important in maintaining cholesterol availability during metamorphic climax. In contrast to cholesterol synthesis, fatty acid elongation and desaturation was increased in metamorphic *R. omeimontis* tadpoles (Fig. 4d). The froglets likely had increased fatty acid unsaturation of the membrane or storage lipids. Metamorphic *R. omeimontis* tadpoles maintained high levels of long-chain polyunsaturated FFAs (e.g., Δ22:5 and Δ22:4) (Fig. 4a). Their increased synthesis in metamorphic tadpoles might be associated with the structural and functional remodeling of the central nervous system at the cellular level [39], as these metabolites are most rich in sphingolipids and are required for neural development [40, 41]. Surprisingly, unsaturated FFA synthesis occurred in the tail, rather than in the liver, during metamorphic climax (Fig. 4e). This suggests a unique role of this apoptotic organ in metabolite interconversion.

Amino acids and monosaccharides are elementary units of proteins and proteoglycans. Both the tail and liver had decreased transcription of genes involved in protein synthesis during metamorphic climax (Fig. 6e). Reduced protein synthesis has also been observed in the liver of metamorphic bullfrog tadpoles [42]. The onset of metamorphic climax involved a transition from growth to differentiation and remodeling [43]. Thus, the intensity of hepatic protein synthesis is determined by the degree of cell type replacement and gene expression switch in the liver. Unlike other organs undergoing remodeling during metamorphic climax (skin, pancreas, and intestine), the liver does not show significant morphological change and cell replacement [26]. Gene expression switching is mainly limited to metabolic enzymes and circulatory proteins (hemoglobin and apolipoprotein), instead of structural components (Fig. 2c and [44]). Accordingly, reduced protein synthesis in the liver is expected during metamorphic climax. Although protein synthesis, an energy-intensive process in hepatocytes [45], was downregulated in the liver of metamorphic tadpoles, the hepatic energy metabolism was upregulated (Additional file 2: Figure S8). It is possible that other energy-intensive biosynthetic pathways were upregulated. Amino acids participate in body construction as protein units, but they also have non-protein functions such as neurotransmitters or precursors of other bioactive metabolites. Glutamine [46] synthesis was upregulated in the tail of metamorphic tadpoles (Fig. 6e and Additional file 2: Figure S7). Glutamine supplements can induce metamorphosis of the sea urchin (*Hemicentrotus pulcherrimus*) [47] and stimulate development in broilers [48]. Its role in amphibian metamorphosis is worthy of further investigation.

The metabolic flux throughout glycolysis was reduced in both the liver and tail of metamorphic tadpoles (Fig. 5c and g). In contrast, the metabolic flux from glucose 1-phosphate to glucose derivatives (glucuronate, UDP-glucosamine, and UDP-glucuronate) was increased (Fig. 5c and g). Glucuronate and glucosamine are required components for the synthesis of mucins and mucopolysaccharides (heparan sulfate and hyaluronan). These biological macromolecules are located at the cell surface and extracellular matrix, [49], and are important in growth and development regulation and morphogenesis in vertebrates [50–54]. Thus, diverting carbohydrates from catabolism to the synthesis of glycan elements might be significant for metamorphosis. In addition, transition to a terrestrial environment involves new environmental stresses, such as dehydration and oxidation. Frog skin has high levels of hyaluronan and mucins [55, 56], which are involved in oxidation resistance and water maintenance [57, 58]. The carbohydrate requirement might be increased during the body construction of froglets. This possibility is supported by the upregulated gluconeogenesis in the liver of metamorphic tadpoles. The increased synthesis of polysaccharides could explain the increased energy metabolism in the liver. The tail and the liver showed increased and decreased metabolic flux throughout PPP, respectively (Fig. 5). This glycol-metabolic pathway plays a critical role in biosynthesis through providing NADPH and carbon skeletons for functional metabolites or structural units of biological macromolecules (DNA and RNA) [31]. Increased flux through PPP is always associated with robust biosynthesis of cellular components [59]. Our results demonstrated that the tail made an increased contribution to metabolite synthesis during metamorphic climax, possibly due to the convenience of metabolic substrates.

### Potential metabolic regulation during metamorphic climax

PPARα is a nutrient-sensing nuclear receptor activating lipolysis, FFA elongation and desaturation, FFA β-oxidation, and hepatic lipid export in the liver and muscle [60–62]. PPARα transcription in the liver and tail was decreased and increased, respectively, during metamorphic climax (Additional file 2: Figure S5). This could explain the decreased hepatic fat mobilization and upregulated tail FFA elongation and desaturation (Figs. 3 and 4). PPARγ is a master regulator of adipocyte differentiation and lipogenesis [63]. Its transcriptional upregulation in the tail was consistent with the encouraged TAG synthesis during metamorphic climax (Fig. 4).

We observed transcription of adiponectin in the tail of *R. omeimontis* tadpoles (Additional file 2: Figure S4). In mammals, this protein hormone is exclusively secreted from adipose tissue into the bloodstream and modulates glucose regulation and fatty acid oxidation in the liver and muscle. It also modulates the adipose tissue in an autocrine/paracrine manner [64, 65]. Although no obvious adipose tissues were observed in the tail of *R. omeimontis* tadpoles [21], it is possible that there are small-scale adipose scatters in this organ. A physiological function of adiponectin is to increase insulin sensitivity by regulating AMPK, PPARα, and p38 MAPK [65]. In our study, decreased expression of adiponectin (Additional file 2: Figure S5) could explain the downregulated fatty acid oxidation and glycolysis, as well as upregulated gluconeogenesis and glycogenolysis, in the liver of metamorphic tadpoles [66, 67]. This might be involved in coordinating the mobilization of hepatic and tail storage. In the tail of metamorphic *R. omeimontis* tadpoles, the transcription of adiponectin receptors was upregulated (Additional file 2: Figure S5), which could off-set the effects of reduced adiponectin on the tail metabolism. However, the transcriptional variation of PPARs and adiponectin did not explain the metabolic adjustments in the tail, potentially due to downregulated metabolism during apoptosis.

A surprising finding here was the increased synthesis of FFA derivatives (e.g., PGs and HEHE) in the tail of metamorphic tadpoles (Fig. 4f–i). These molecules have proinflammatory and anti-inflammatory activities [68, 69]. They have been identified as ligands of PPARs [70–72] and their increased synthesis might be involved in the metabolic regulation in a PPAR dependent manner. These lipids also play important roles in development and apoptosis [73–76]. It is possible they also have a role in regulating the metamorphic climax. Further studies may reveal their physiological and metabolic functions.

## Conclusion

**Fig. 7:**
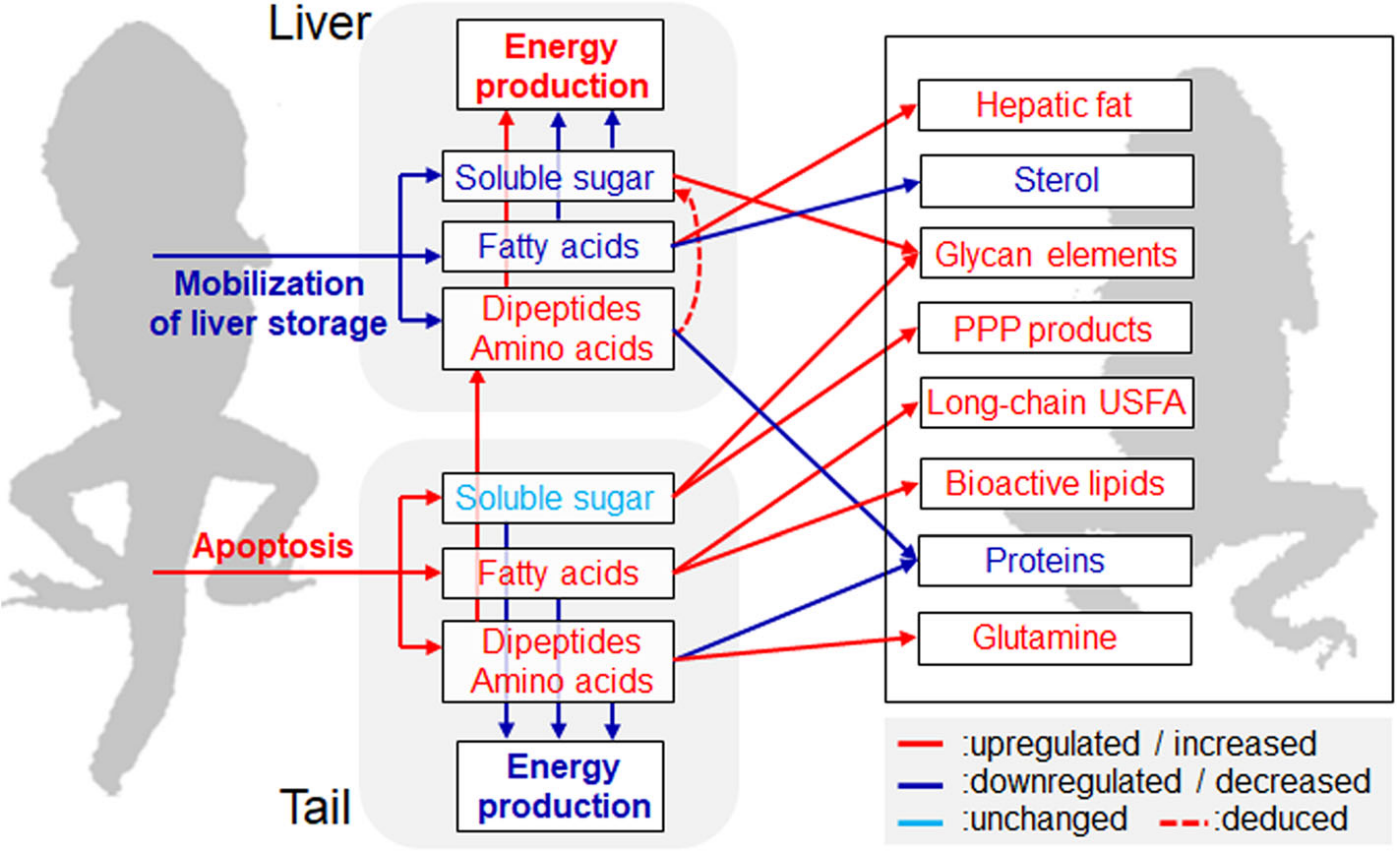
Variation of metabolic fluxes during metamorphic climax. Items and arrows with blue, red, and cyan colors mean downregulated/decreased, upregulated/increased, and unchanged, respectively. The thickness of arrows is uniform, and it does not indicate the level of metabolic flux. Arrows in dashed lines indicates deduced adjustment on this metabolic pathway

We reconstructed the lipid, carbohydrate, and amino acid metabolic networks and analyzed the metabolic adjustments during the onset of metamorphic climax in *R. omeimontis* tadpoles (Fig. 7). We showed (1) the energy requirement and metabolic switch during the onset of metamorphic climax; (2) the anabolic requirements in metamorphic tadpoles, especially the increased synthesis of glycan elements and unsaturated FFAs; (3) the contribution of the apoptotic tail to anabolism. These findings illustrate the metabolic requirements of amphibian metamorphosis. They may relate to ecological, toxicological, and developmental studies using amphibians as a model system.

## Supplementary information

**Additional file 1: **Liver and tail metabolites tables. **Table S1.** Liver metabolomes of stage 36, 41, 43, and 44 tadpoles. **Table S2.** Tail metabolomes of stage 36, 41, and 43 tadpoles. **Table S3.** Liver metabolomes of control and T3-treated stage 30–31 tadpoles. **Table S4.** Tail metabolomes of control and T3-treated stage 30–31 tadpoles. **Table S5.** Results of KEGG enrichment analyses based on liver DEGs between control and T3-treated tadpoles. **Table S6.** Results of KEGG enrichment analyses based on tail DEGs between control and T3-treated tadpoles.

**Additional file 2: Figure S1.** Overview of the transcriptomes of control and T3-treated tadpoles. (A) Unigene length distribution. (B) Gene expression correlations between samples. (C–D) Volcano plots showing the DEGs of liver (C) and tail (D) metabolomes between control and T3-treated tadpoles. **Figure S2.** Liver size of control and T3-treated tadpoles. ***, *p* < 0.001. **Figure S3.** Scatter plots of PCAs based on liver (A) and tail (B) metabolomes of pro-metamorphic (stage 30–31) and T3-driven metamorphic tadpoles. **Figure S4.** Transcriptional variation of genes involved in primary bile acid biosynthesis and steroid metabolism in the liver. *, *p* < 0.05; **, *p* < 0.01; ***, *p* < 0.001. **Figure S5.** Transcriptional variation of genes with potential metabolic regulatory functions in the liver and tail. *, *p* < 0.05. **Figure S6.** Arachidonic acid metabolism highlighted by tail DEGs between control and T3-treated tadpoles. **Figure S7.** Variation of glutamine levels during metamorphic climax. Different letters denote significant differences between groups (*p* < 0.05), as shown by the Student**–**Newman**–**Keuls post hoc test after one-way ANOVA. *, *p* < 0.05. **Figure S8.** Transcriptional changes of genes involved in energy metabolism during metamorphic climax. (A) Transcriptional changes of genes involved in energy metabolism (TCA cycle and oxidative phosphorylation) in the liver. (B) Transcriptional changes of genes involved in TCA cycle in the tail. (C) Heatmap showing the transcriptional level of genes involved in oxidative phosphorylation in the tail. (D) Transcriptional changes of major energy consuming proteins in the tail. A positive logarithmic transformed fold change value means upregulation in T3-treated group, and vice versa; *, *p* < 0.05. PDH, pyruvate dehydrogenase; CS, citrate synthase; IDH, isocitrate dehydrogenase; 2-OGDH, 2-oxoglutarate dehydrogenase; SCL, succinyl-CoA ligase; SDH, succinate dehydrogenase; MDH, malate dehydrogenase; ND, NADH dehydrogenase; MCK, muscle creatine kinase; UCP, uncoupling protein.

## Data Availability

The sequencing data from this study were submitted to the NCBI Gene Expression Omnibus (GEO; http://www.ncbi.nlm.nih.gov/geo/) under accession number GSE147618.
