# Supplementary material for: Remarkable metabolic reorganization and altered metabolic requirements in frog metamorphic climax
Source: Front Zool. 2020 Oct 8;17:30. doi: 10.1186/s12983-020-00378-6 (PMC7542913; doi:10.1186/s12983-020-00378-6)
Supplement: Supplementary file 2 — Additional file 2: Figure S1. Overview of the transcriptomes of control and T3-treated tadpoles. (A) Unigene length distribution. (B) Gene expression correlations between samples. (C–D) Volcano plots showing the DEGs of liver (C) and tail (D) metabolomes between control and T3-treated tadpoles. Figure S2. Liver size of control and T3-treated tadpoles. ***, p < 0.001. Figure S3. Scatter plots of PCAs based on liver (A) and tail (B) metabolomes of pro-metamorphic (stage 30–31) and T3-driven metamorphic tadpoles. Figure S4. Transcriptional variation of genes involved in primary bile acid biosynthesis and steroid metabolism in the liver. *, p < 0.05; **, p < 0.01; ***, p < 0.001. Figure S5. Transcriptional variation of genes with potential metabolic regulatory functions in the liver and tail. *, p < 0.05. Figure S6. Arachidonic acid metabolism highlighted by tail DEGs between control and T3-treated tadpoles. Figure S7. Variation of glutamine levels during metamorphic climax. Different letters denote significant differences between groups (p < 0.05), as shown by the Student–Newman–Keuls post hoc test after one-way ANOVA. *, p < 0.05. Figure S8. Transcriptional changes of genes involved in energy metabolism during metamorphic climax. (A) Transcriptional changes of genes involved in energy metabolism (TCA cycle and oxidative phosphorylation) in the liver. (B) Transcriptional changes of genes involved in TCA cycle in the tail. (C) Heatmap showing the transcriptional level of genes involved in oxidative phosphorylation in the tail. (D) Transcriptional changes of major energy consuming proteins in the tail. A positive logarithmic transformed fold change value means upregulation in T3-treated group, and vice versa; *, p < 0.05. PDH, pyruvate dehydrogenase; CS, citrate synthase; IDH, isocitrate dehydrogenase; 2-OGDH, 2-oxoglutarate dehydrogenase; SCL, succinyl-CoA ligase; SDH, succinate dehydrogenase; MDH, malate dehydrogenase; ND, NADH dehydrogenase; MCK, muscle creatine kinas [file 12983_2020_378_MOESM2_ESM.pdf]

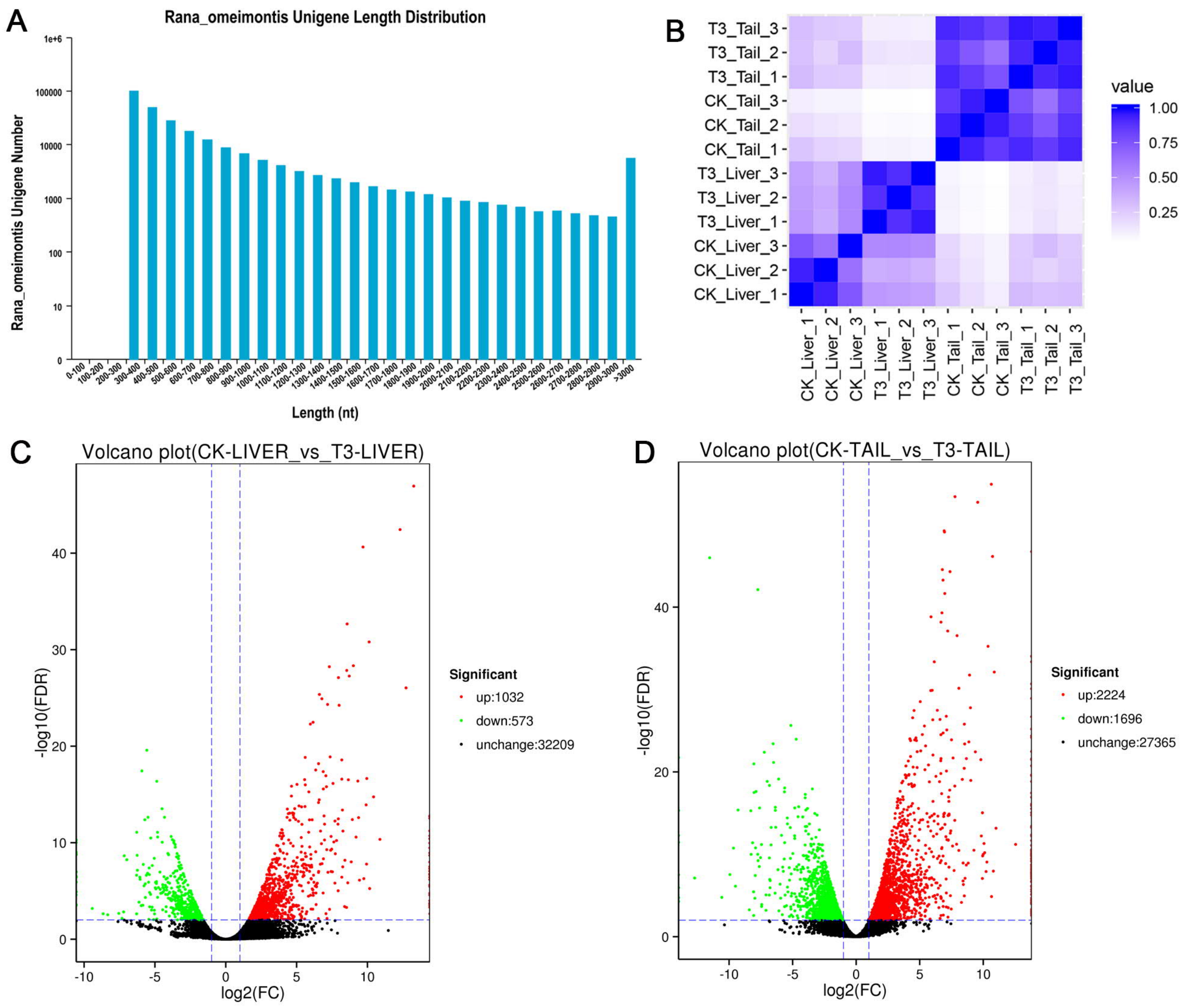

Figure S1. An overview of the transcriptomes of control and T3-treated tadpoles. (A) Unigene length distribution. (B) Gene expression correlations between samples. (C-D) Volcano plots presenting the DEGs of liver (C) and tail (D) metabolomes between control and T3-treated tadpoles.

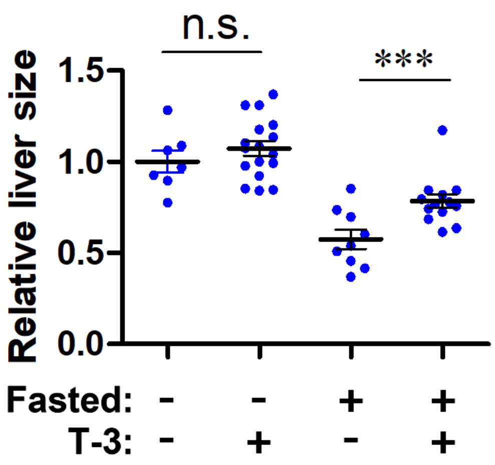

Figure S2. Liver size of control and T3-treated tadpoles. ***, $p < 0.001$.

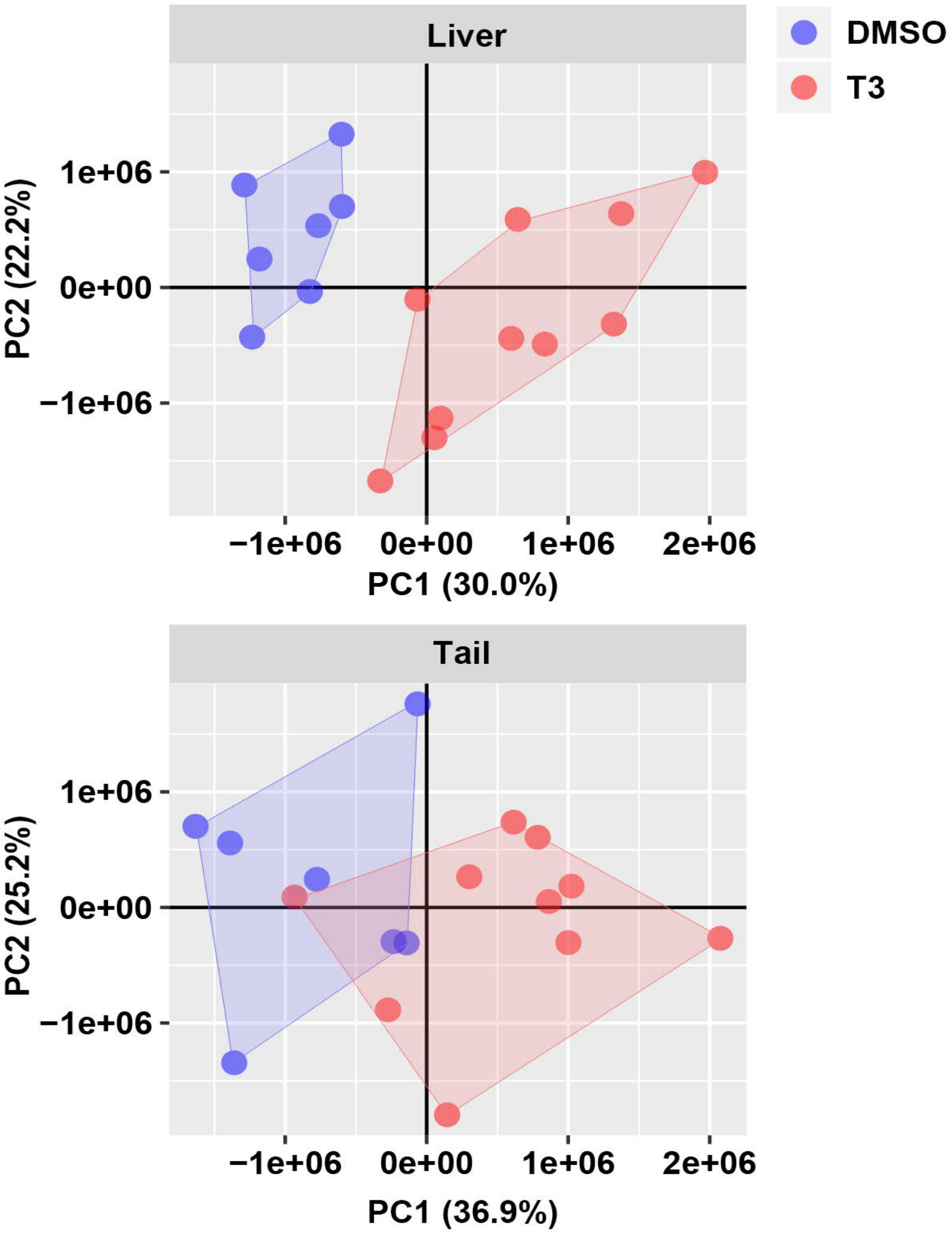

Figure S3. Scatter plots of PCAs based on liver (A) and tail (B) metabolomes of prometamorphic (stage 30-31) and T3-driven metamorphic tadpoles.

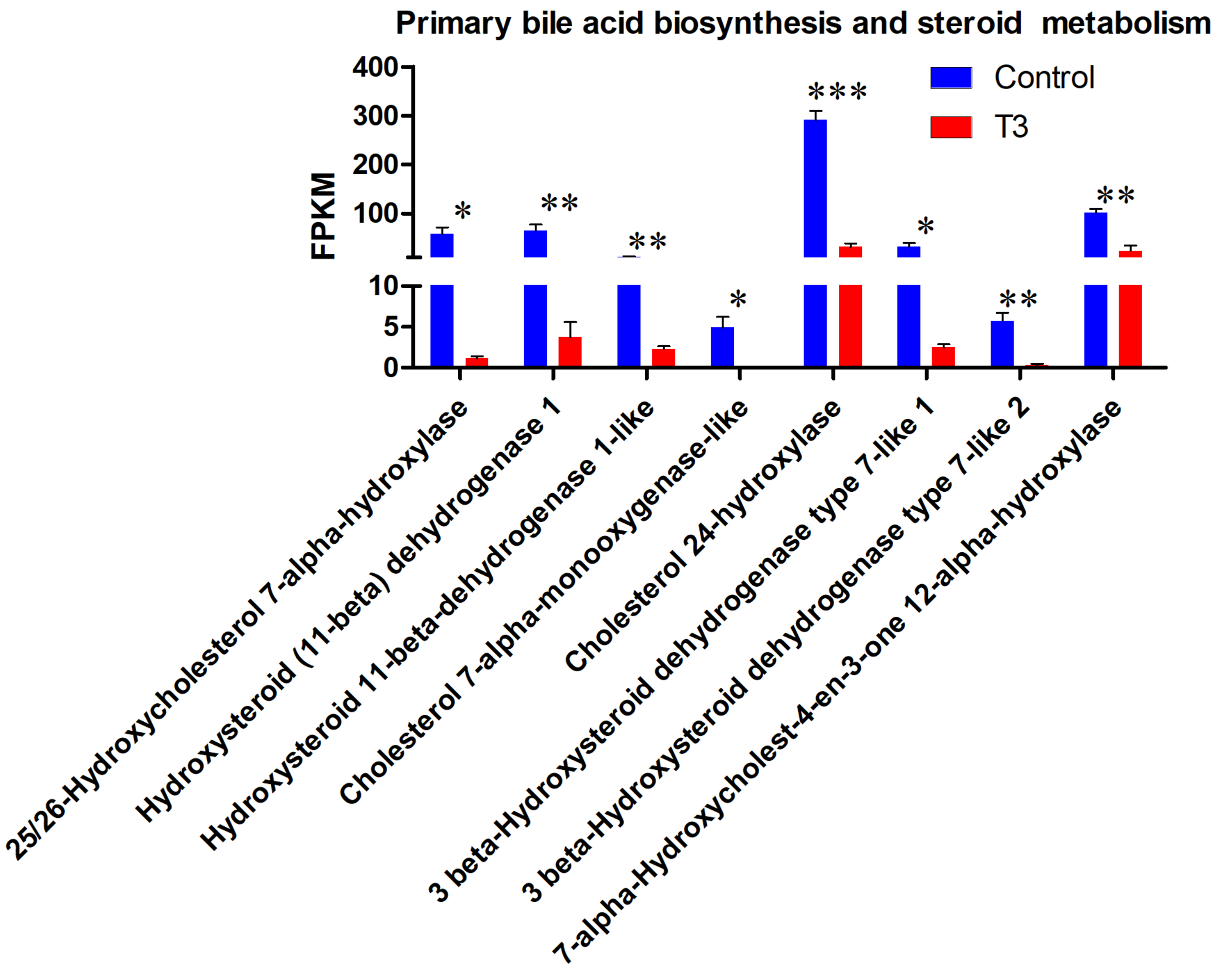

Figure S4. Transcriptional variation of genes involved in primary bile acid biosynthesis and steroid metabolism in the liver. *, $p < 0.05$; **, $p < 0.01$; ***, $p < 0.001$.

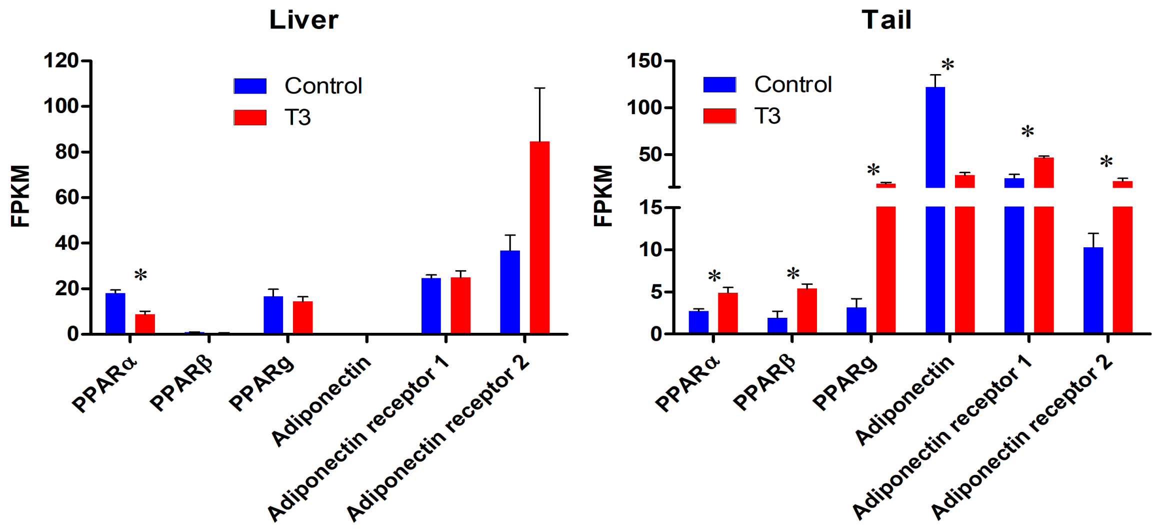

Figure S5. Transcriptional variation of genes with potential metabolic regulatory functions in the liver and tail. *, $p < 0.05$.

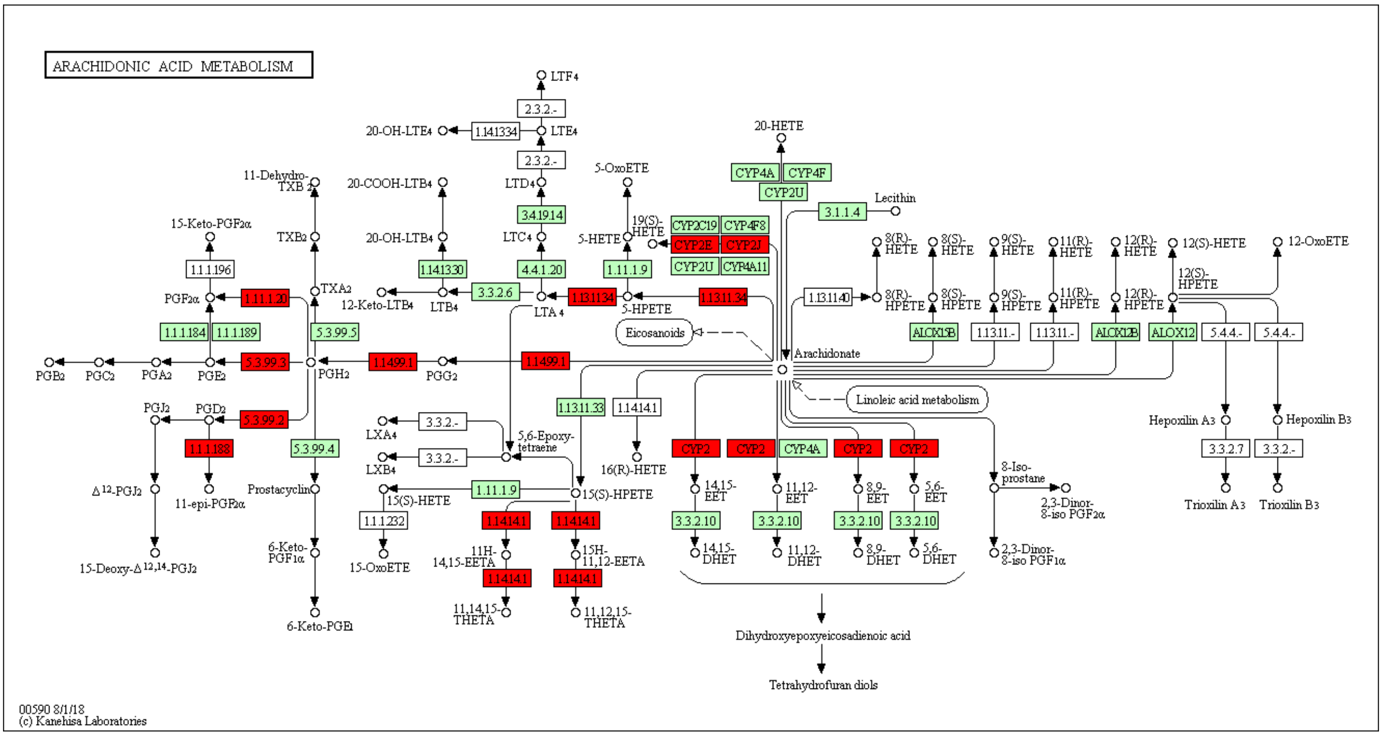

Figure S6. Arachidonic acid metabolism highlighted by tail DEGs between control and T3-treated tadpoles.

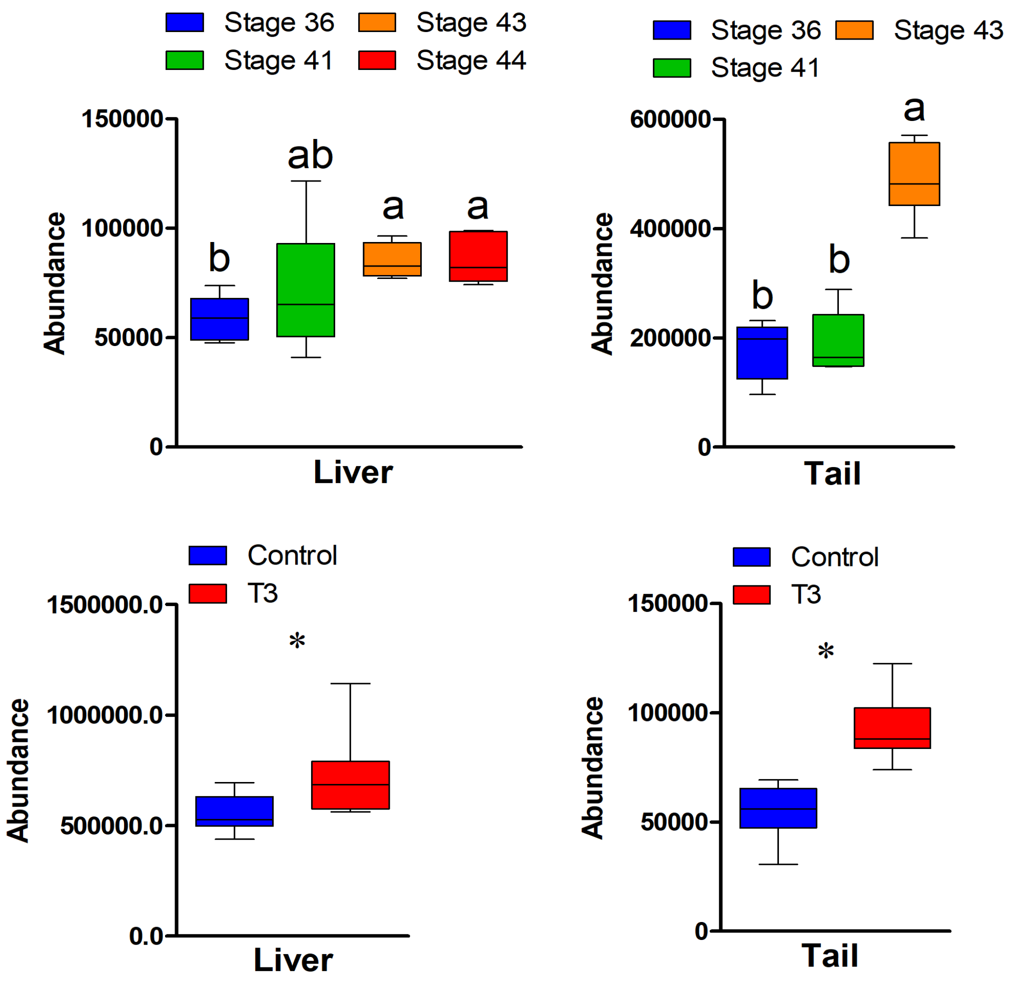

Figure S7. Variation of glutamine level during metamorphic climax. Different letters denote significant differences between groups ($p < 0.05$), as shown by the Student Newman Keuls post hoc test after one-way ANOVA. *, $p < 0.05$.

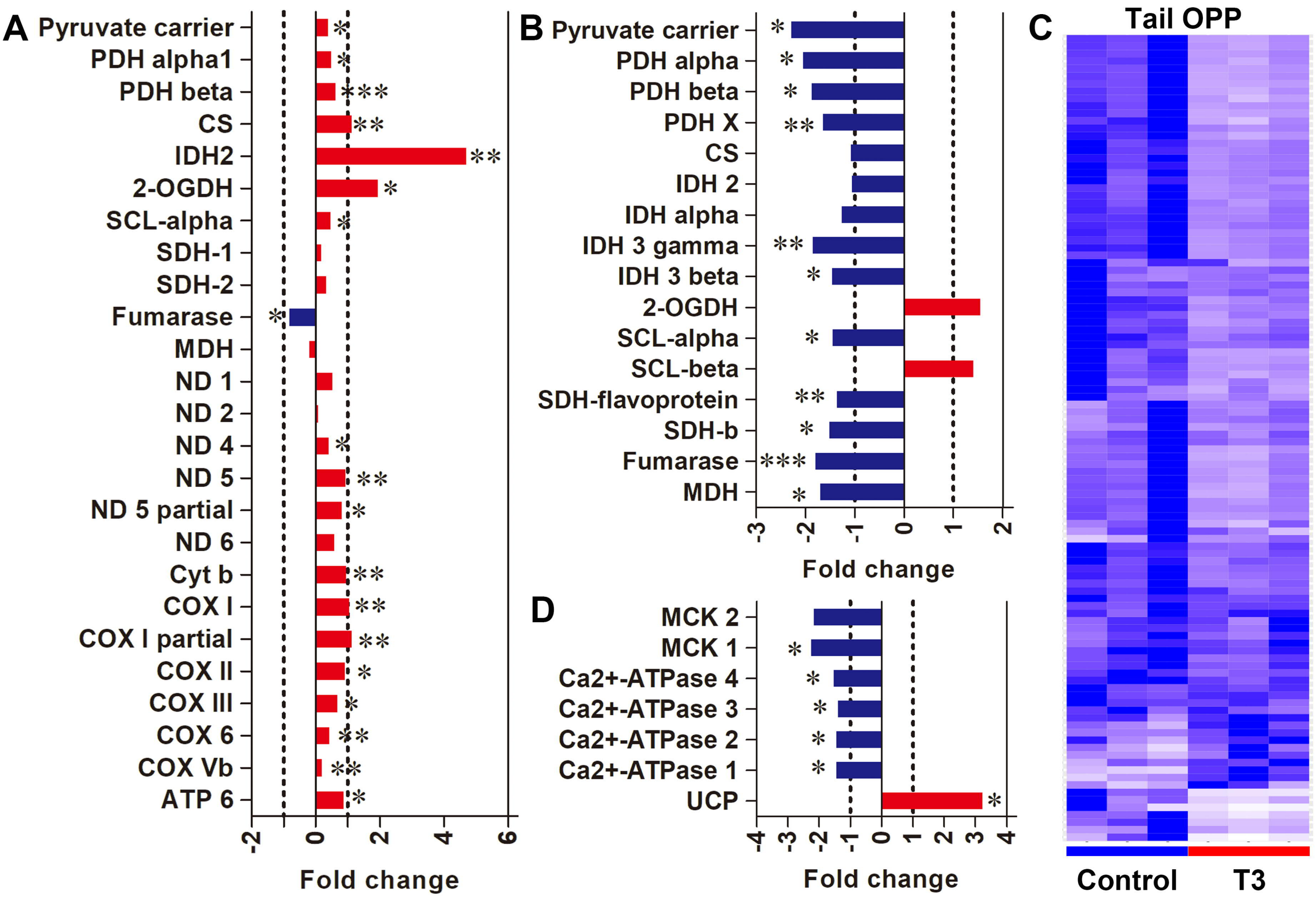

Figure S8. Transcriptional changes of genes involved in energy metabolism during metamorphic climax. (A) Transcriptional changes of genes involved in energy metabolism (i.e., TCA cycle and oxidative phosphorylation) in the liver. (B) Transcriptional changes of genes involved in TCA cycle in the tail. (C) Heatmap presenting the transcriptional level of genes in oxidative phosphorylation in the tail. (D) Transcriptional changes of major energy consuming protein in the tail. A positive logarithmic transformed fold change value means upregulation in T3-treated group, and vice versa; *, $p < 0.05$. PDH, pyruvate dehydrogenase; CS, citrate synthase; IDH, isocitrate dehydrogenase; 2-OGDH, 2-oxoglutarate dehydrogenase; SCL, succinyl-CoA ligase; SDH, succinate dehydrogenase; MDH, malate dehydrogenase; ND, NADH dehydrogenase; MCK, muscle creatine kinase; UCP, uncoupling protein.
